# Awareness and Use of Virtual Clinics following the COVID-19 Pandemic in Saudi Arabia

**DOI:** 10.3390/healthcare10101893

**Published:** 2022-09-28

**Authors:** Saja Al-Rayes, Arwa Alumran, Haifa Aljanoubi, Aram Alkaltham, Manar Alghamdi, Duaa Aljabri

**Affiliations:** Department of Health Information Management and Technology, College of Public Health, Imam Abdulrahman Bin Faisal University, Dammam 34212, Saudi Arabia

**Keywords:** virtual clinics, awareness, use, post COVID-19, Technology Acceptance Model (TAM), usefulness, ease of use, Saudi Arabia

## Abstract

Studies have shown that virtual clinics enjoyed high use and high patient satisfaction during the COVID-19 pandemic. Thus, virtual clinics are expected to be the new normal mode of receiving care after the COVID-19 pandemic. This study aimed to assess public awareness and use of virtual clinics following the pandemic and identify factors associated with virtual clinic use. Methods: A cross-sectional design was employed in which data were collected via a structured online questionnaire based on the Technology Acceptance Model (TAM) domains: perceived usefulness, perceived ease of use, and social influence. Participants were selected based on the non-probability sampling of convenience. Univariate, bivariate, and binary logistic regression models were used for analysis. Results: A total of 405 responses were received; of those, 286 (70.6%) were aware of the existence of virtual clinics and 99 (34.6%) were post-pandemic users. Among users, 50% used virtual clinics more than two times, 72% used virtual clinics to seek care for themselves, with the vast majority using it via voice calls (83.8%), and for visits to the family medicine clinic (55%). Young adults, females, single adults, those with a higher level of education, the employed, and those with lower income were more likely to use virtual clinics (*p* < 0.05). The logistic regression model showed that 20% of the variation in virtual clinic use was explained by perceived usefulness and perceived use (*p* < 0.001). Conclusion: This study showed high awareness of virtual clinics among the population following the COVID-19 pandemic, with one-third being active users. Age, gender, marital status, education, income, employment status, perceived usefulness, and ease of use are associated with virtual clinics’ awareness and use. Considering those factors is important when planning for sustained use of e-health and virtual care.

## 1. Introduction

The healthcare system is evolving at a rapid pace as healthcare providers look for ways to increase healthcare quality and accessibility while decreasing the overall cost. A virtual clinic is a digital technology that allows remote and real-time interaction between patients and doctors through telephone or video calls for diagnosis, examination, and medical assessment [1]. Virtual care can be defined as any remote interaction between patients and healthcare providers, using any form of communication or information technology, to facilitate or maximize the quality and effectiveness of patient care [2].

Several studies [1,2,3,4] have discussed the benefits of virtual clinics, such as saving time and money on in-person visits and reducing the cost of transportation. In addition, convenience and accessibility are key for patients living in remote areas or those who cannot afford to take time off work. Others found virtual care useful in preventing the spread of infections by keeping patients out of the waiting room. Thus, virtual clinics have gained a high level of satisfaction and trust among the population, despite the service being relatively new [5]. McGrail et al. indicated that 93% stated that the virtual visit was of high quality and 91% indicated that the virtual visit was useful in resolving their health issues, indicating high patient satisfaction [6]. Additionally, others indicated that, during the pandemic, the quality of virtual clinic service was comparable to actual office visits with the additional benefit of fast access to care [7,8].

As part of the Saudi vision 2030 framework released in 2017, the path for digital health transformation initiatives was paved and the use of informatics tools has bloomed since the COVID-19 pandemic [9]. Specifically, the use of virtual clinics has increased dramatically since the onset of the pandemic in 2020 as one of the strategies to support public health control measures in reducing the risk of disease transmission and minimizing exposure to health facilities [10]. The delivery of virtual clinics in Saudi Arabia is provided through several mechanisms. Firstly through hospital appointments, where the doctor determines whether or not the patient requires his presence, or if the patient wishes to obtain the service remotely. Secondly through the Sehhaty mobile application, where the patient can directly book an appointment if the service is available in the healthcare facility. Since February 2022, virtual clinics expanded with the launching of “Seha Virtual Hospital”, which is considered the first virtual hospital in the Middle East and the largest virtual hospital in the world. The hospital supports a total of 130 hospitals around the country and provides specialized virtual clinics such as psychiatry, cardiac, endocrine, and diabetes clinics [11]. In addition, virtual multidisciplinary committees composed of multiple consultants and experts from various health facilities were grouped and enabled to communicate and provide their specialized opinions based on the patient’s case. The capacity of the hospital during the year reaches over 400 thousand beneficiaries. Given the rising expectations to continue adopting virtual care, there are limited data that explore public utilization of virtual care following the pandemic. This study evaluates public awareness and use of virtual clinics following the pandemic and identifies the factors affecting the actual use among the general adult population. Understanding those factors will help inform the digital strategy as it matures and support the design of plans for sustained virtual clinic use. This has the potential to improve access and continuity of care to patients.

## 2. Study Theoretical Model

Many theories exist in social science to predict human behavior, such as the Health Belief Model [12], Social Cognitive Theory [13], Theory of Reasoned Action [14], and Theory of Planned Behavior [15]. However, the Technology Acceptance Model (TAM) is one of the well-known theories to predict human behavior in the adoption of new technologies, such as virtual clinics [16,17]. Based on TAM, perceived usefulness (PU) and perceived ease of use (PEU) are the two main predictors of the intention of technology use. PU refers to the level to which someone thinks that it would be beneficial to use new technology in terms of providing timely and low-cost care. PEU refers to the level to which someone thinks that it would be effortless to use new technology. TAM suggests that, the higher the PU and PEU of a technology a person has, the higher the probability of that person’s intent to use the technology.

In addition, other variables from the literature are found to be associated with technology use such as social influence (SI) and demographic variables. SI refers to the level to which someone thinks that others, particularly his/her friends and acquaintances, believe that he/she should use a new system [18]. In Saudi Arabia, there was a significant association between SI and the use of technology in the health sector, including electronic health records [19], electronic triage systems [20], and mobile healthcare services [21]. Furthermore, a study from Pakistan showed that SI affected the public use of telemedicine [18]. Similarly, researchers from China proved that patients’ adoption of telemedicine was influenced by their social environments [22]. Therefore, we measured the SI and its impact on the use of virtual clinics. Moreover, scholars found that the use of telemedicine was determined by the user’s demographics involving age [23,24], gender [23,24], marital status [23], income [23,25], and education level [25]. Thus, this study also assessed the influence of demographic variables on the use of virtual clinics.

## 3. Material and Methods

### 3.1. Study Design and Population

This paper presents a cross-sectional study conducted in March 2022 among the general adult population aged 18 years or older residing in Saudi Arabia. Participants were selected using a nonprobability sampling technique, namely convenience sampling. To achieve a 95% confidence interval with a 5% margin of error, the minimum required sample size for large populations was 384 [26].

### 3.2. Survey Instrument

A structured online anonymous questionnaire was used for data collection. The questionnaire consisted of 31 questions organized into three sections. The first section included two questions about awareness (aware, unaware) and use (user, non-user) of virtual clinics, with seven follow-up questions asked to users (i.e., frequency of visits, type of visit, the visitor, type of clinic, type of healthcare facility, mode of communication, and the device used in their last visit to the virtual clinic). The second section included twelve questions informed by elements from the Technology Acceptance Model, with four questions per construct: PU [18,27,28], PEU [18,28], and SI [29]. Questions were adopted from previous studies with few modifications to suit the context and measured on a five-point Likert scale, ranging from “strongly disagree” = 1 to “strongly agree” = 5. The sum of all points answered per construct was calculated and analysed, where a higher score of a construct indicates a greater likelihood of using virtual clinics, and vice versa. The third section of the questionnaire included demographic questions such as gender, nationality, age, marital status, residential region, education level, occupational status, monthly income, and the existence of chronic diseases (Supplementary file S1).

### 3.3. Instrument Validation

Linguistic validity was conducted using the translate–translate back methodology, in which two independent certified translation offices translated the questionnaire from English to Arabic and back into English [30]. Face and content validity of the questionnaire was ensured by consultation with expert faculty members at Imam Abdulrahman Bin Faisal University (IAU) and then pilot-tested with a sample of individuals from the general public of Saudi Arabia (i.e., the intended sample) [30]. The internal consistency reliability was tested through Cronbach’s alpha (PU = 0.823, PEU = 0.807, and SI = 0.859). The testing revealed that each factor had a Cronbach’s alpha higher than 0.8, indicating a good internal consistency [31].

### 3.4. Statistical Analysis

Univariate analysis was conducted using frequencies and percentages for categorical variables, means, SD, and ranges for continuous variables [32]. Skewness and kurtosis criteria were used to test the normality of the continuous variables and showed a normal distribution [32]. Bivariate analysis was conducted using Chi-square tests and Fisher’s exact tests to examine the factors affecting virtual clinic use. Multivariable analysis using binary logistic regression was performed for model testing. All analyses were completed using IBM SPSS Statistics (Version 26.0. Armonk, NY, USA: IBM Corp) [33]. A *p*-value less than 0.05 was considered statistically significant.

## 4. Results

### 4.1. Descriptive Statistics (Univariate Analysis)

A total of 405 individuals participated in this study. Most participants were females (*n* = 299, 73.8%), between 18 and 30 years of age (*n* = 287, 70.9%), not married (*n* = 280, 69.1%), Saudi citizens (*n* = 388, 95.8%), and residing in the Eastern province of Saudi Arabia (*n* = 293, 72.3%). More than half of the respondents indicated that they were unemployed (*n* = 249, 61.5%) and the average monthly income in the study group was less than 2801.12 USD (*n* = 239, 59%). Almost half of the study participants had a bachelor’s degree (*n* = 192, 47.4%) and most of the participants indicated that they did not have any chronic disease (*n* = 348, 85.9%) (Table 1).

### 4.2. Virtual Clinic Users

Among users, 49.5% used virtual clinics more than two times. In addition, in their last visit to virtual clinics, 54.5% of users reported that they went for an initial consultation, 72.7% used virtual clinics to seek care for themselves, and most visits were to the family medicine clinic (55.6%). Furthermore, 76.8% of users went for a governmental hospital in their last virtual clinic visit, with the vast majority using it via voice calls (83.8%) via their cellphones (94%) (See Table 2).

### 4.3. Bivariate Analysis

Table 3 shows the factors that influence the utilization of virtual clinics. Gender was significantly associated with virtual clinic use (x^2^ = 6.037, *p* = 0.014). The majority of females reported that they have not used virtual clinics before (*n* = 154, 69.1% of all females). Age was significantly associated with the actual use of virtual clinics (x^2^ = 11.323, *p* = 0.01). Among adults between 18 and 30 years old, 143 (71.1%) individuals were non-users. Marital status also significantly influences respondents’ use (x^2^ = 10.714, *p* = 0.001). Most unmarried individuals were non-users of virtual clinics (*n* = 141, 71.6% of all unmarried participants).

Participants’ educational level significantly influences their virtual clinic use (x^2^ = 12.349, *p* = 0.006). Those with a bachelor’s degree or lower tend to be non-users. In addition, occupational status was also found to significantly influence virtual clinic utilization (x^2^ = 10.969, *p* = 0.003). Most unemployed individuals have never used a virtual clinic (*n* = 128, 72.7% of all unemployed participants), while 46% of the employed participants indicated that they have used a virtual clinic at least once (*n* = 46). Furthermore, monthly income was significantly associated with the actual use of virtual clinics (x^2^ = 12.501, *p* = 0.014). Participants with a monthly income of less than 2801.12 USD were more likely to be non-users (*n* = 118, 70.7%). On the other hand, 60.6% of those with a monthly income between 8403.92 and 11,204.48 USD were actual users of virtual clinics (*n* = 20). Nationality, living region, and chronic diseases did not have any significant influence on participants’ actual use of the virtual clinic (Table 3).

Table 4 shows the TAM domains associated with the actual use of the virtual clinic. The table shows that all domains are significantly related to the actual use of the virtual clinic (PU: *t* = 2.558, *p* = 0.011; PEU: *t* = 6.627, *p* < 0.001; and SI: *t* = 3.707, *p* < 0.001). All users of the virtual clinics have on average higher scores in all domains.

### 4.4. Interpretation of Multivariable Analysis

**Figure 1:**
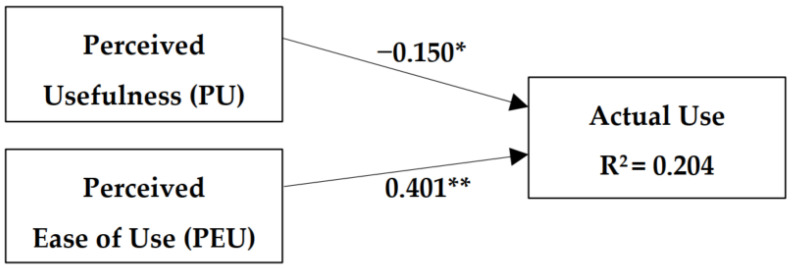
Model 1: The model includes perceived usefulness and perceived ease of use as independent variables. Both variables were found to be associated with individuals’ use of virtual clinics. The model explained 20.4% of the actual use of virtual clinics and the beta for both variables was significant. * *p* < 0.05; ** *p* < 0.001.

Using a binary logistic regression, two models were tested to predict the actual use of virtual clinics (Table 5). The first model contains PU and PEU as the independent variables. As shown in Figure 1, this model explained 20.4% of the actual use of virtual clinics, and the beta for both variables was significant.

**Figure 2:**
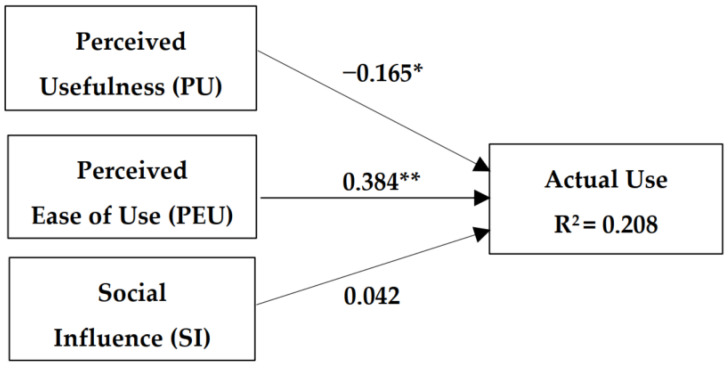
Model 2: The model includes perceived usefulness, perceived ease of use, and social influences as independent variables. The presented model explained 20.8% of the actual use of virtual clinics. However, the beta for the SI variable was not statistically significant. * *p* < 0.05; ** *p* < 0.001.

The second model contains PU, PEU, and SI as the independent variables. As shown in Figure 2, this model explained 20.8% of the actual use of virtual clinics, and the beta for the SI variable was not significant. As there was no major increase in the explanatory power (R2) after the addition of SI, the effect of SI was not significant.

## 5. Discussion

The study’s main objective was to assess public awareness and use of virtual clinics following the pandemic and to identify factors associated with virtual clinic use. The findings showed that 70.6% of participants were aware of the existence of virtual clinics and 34.6% of participants were post-pandemic users. Young adults, females, single adults, those with a higher level of education, the employed, and those with lower income were more likely to use virtual clinics. Those factors were in accordance with previous literature that shows an association between using virtual care and sociodemographic characteristics such as age [23,24], gender [23,24], marital status [23], income [23,25,34], and education level [25].

User-friendly digital tools and interfaces play an important role in the use of virtual care [35]. The perceived usefulness and ease of use reported in this study were significant predictors of virtual clinic use. Our findings are consistent with Kamal et al. (2020), who found that ease, satisfaction, and comfort in using virtual care devices are associated with such use [18]. Yet, social influence was not a significant predictor of the use of virtual clinic use in our model. This finding contradicts the results of an earlier study reporting that social influence was a strong explanatory factor for the use of electronic health record systems in Saudi Arabia [19]. However, this contradiction could be due to the different time frames in which both studies were conducted as well as the intended sample.

As usefulness and ease of use were reported to be key factors affecting virtual clinic use, healthcare leaders must focus on those features in their future improvement plans. It is essential to note that most reported worldwide barriers include digital infrastructure limitations and technology-specific issues such as limited internet connectivity or speed as well as risks related to data security [36]. Other patient-specific barriers include a lack of access to proper communication equipment and patients’ low digital health literacy [35,37]. Those barriers can be eliminated by focused policy to strengthen the digital infrastructure and engage all relevant stakeholders to adequately adopt this technology. In addition, other strategies are needed to enhance the user interface design, improve communication between providers and patients, and teach both providers and patients the technological skills required to access virtual clinics. Incentives can also be considered to overcome barriers and encourage using virtual care, which can take the form of free or lower fee-for-service for patients and monetary incentives for providers. Most importantly, regulating virtual clinic platforms and developing standalone virtual clinic practical guidelines are essential to govern providing and receiving virtual care [2].

This study is the first to measure public awareness and the use of virtual clinics following COVID-19 in Saudi Arabia, and to identify factors affecting the use of virtual clinics in light of the Technology Acceptance Model. However, it has limitations. First, the sampling technique was nonprobability sampling, which limits our ability to generalize the results of this paper because of the lack of random selection of participants. Furthermore, the sample size was unequal as it included nearly two-thirds of females, and most participants were aged 18–30 years old and lived in the eastern province. This could be because the study questionnaire was carried out by authors located in the eastern province. In addition, data were distributed using an online survey on social media, which limited measuring the response rate and making the data subject to technological literacy bias.

## 6. Conclusions

The study showed high awareness of virtual clinics among the population following the COVID-19 pandemic, with one-third being active users. The study shed light on factors associated with virtual clinic use, which need to be considered to sustain the use of virtual care. Investing in enhancing the design of digital tools and platforms to be user-friendly and easy to use is important to engage patients in using virtual clinics, which has the potential to improve accessibility and continuity of care.

## Figures and Tables

**Table 1:** Sample profile.

Variables		*N* (%)
Awareness of virtual clinics (*n* = 405)	Aware	286 (70.6)
	Not aware	119 (29.4)
Users of virtual clinics (*n* = 286)	Yes	99 (34.6)
	No	187 (65.3)
**Sample Characteristics (*n* = 405)**		***N* (%)**
Gender	Male	106 (26.2)
	Female	299 (73.8)
Nationality	Saudi	388 (95.8)
	Non-Saudi	17 (4.2)
Age (in years)	18–30	287 (70.9)
	31–40	51 (12.6)
	41–50	30 (7.4)
	50+	37 (9.1)
Marital status	Single	280 (69.1)
	Married	125 (30.9)
Residential region	Eastern	293 (72.3)
	Central	45 (11.1)
	Western	38 (9.4)
	Northern	5 (1.2)
	Southern	24 (5.9)
Education level	Highschool or less	128 (31.6)
	Diploma	27 (6.7)
	Bachelor	192 (47.4)
	Postgraduate	58 (14.3)
Employment status	Employed	137 (33.8)
	Unemployed	249 (61.5)
	Retired	19 (4.7)
Monthly income	≤2801.12 USD	239 (59.0)
	2801.68–5602.24 USD	44 (10.9)
	5602.80–8403.36 USD	43 (10.6)
	8403.92–11,204.48 USD	42 (10.4)
	>11,204.48 USD	37 (9.1)
Existence of chronic disease	Yes	57 (14.1)
	None	348 (85.9)

**Table 2:** Descriptive statistics for follow-up questions asked to virtual clinics users (*n* = 99).

Questions		*N* (%)
How many times did you use the virtual clinic?	Once	24 (24.2)
	Twice	26 (26.3)
	More than 2 times	49 (49.5)
**In your last visit to the virtual clinic:**		
What was the type of your visit?	Initial consultation	54 (54.5)
	Follow up	45 (45.5)
Who was the visit for?	Self	72 (72.7)
	Else	27 (27.3)
What was the clinic type?	Family medicine	55 (55.6)
	Internal medicine	4 (4)
	Pediatric	8 (8.1)
	Obstetrics and gynecology	3 (3)
	Ophthalmology	1 (1)
	Dermatology	6 (6.1)
	Ear, nose, and throat	6 (6.1)
	Psychiatry	8 (8.1)
	Neurology	1 (1)
	Urology	2 (2)
	Dentistry	3 (3)
	Other	2 (2)
What was the type of the healthcare facility?	Private	23 (23.2)
	Governmental	76 (76.8)
What was the mode of communication?	Voice call	83 (83.8)
	Video call	16 (16.2)
What type of device did you use?	Cell phone	93 (94)
	Tablet device	3 (3)
	Laptop	3 (3)

**Table 3:** Sociodemographic factors affecting virtual clinics’ use.

Variable	Have You Ever Used Virtual Clinics?		*p*-Value
	Yes (*n* = 99) *N* (%)	No (*n* = 187) *N* (%)	
Gender			**(0.014)** ^a^
Male	30 (30.3)	33 (17.6)	
Female	69 (69.7)	154 (82.4)	
Nationality			(0.935) ^b^
Saudi	96 (97.0)	181 (96.8)	
Non-Saudi	3 (3.0)	6 (3.2)	
Age			**(0.010)** ^a^
18–30	58 (58.6)	143 (76.5)	
31–40	22 (22.2)	21 (11.2)	
41–50	12 (12.1)	11 (5.9)	
>50	7 (7.1)	12 (6.4)	
Marital status			**(0.001)** ^a^
Single	56 (56.6)	141 (75.4)	
Married	43 (43.4)	46 (24.6)	
Education level			**(0.006)** ^a^
High school or less	21(21.2)	68 (36.4)	
Diploma	7 (7.1)	13 (7)	
Bachelor	44 (44.4)	82 (43.9)	
Postgraduate	27 (27.3)	24 (12.8)	
Employment status			**(0.003)** ^b^
Employed	48 (48.5)	54 (28.9)	
Unemployed	46 (46.5)	128 (68.4)	
Retired	5 (5.1)	5 (2.7)	
Monthly income			**(0.014)** ^a^
≥2801.12 USD	49 (49.5)	118 (63.1)	
2801.68–5602.24 USD	9 (9.1)	19 (10.2)	
5602.80–8403.36 USD	10 (10.1)	21 (11.2)	
8403.92–11,204.48 USD	20 (20.2)	13 (7)	
<11,204.48 USD	11 (11.1)	16 (8.6)	
Have a chronic disease			(0.117) ^a^
Yes	19 (19.2)	23 (12.3)	
No	80 (80.8)	164 (87.7)	

^a^ Chi-square test; ^b^ Fisher exact. Bold values indicate significant association.

**Table 4:** TAM factors affecting virtual clinics’ use.

Variable	Have You Ever Used Virtual Clinics? Mean (SD)		Mean Difference (95% CI)	*t*-Test (*p*-Value)
	Yes (*n* = 99) *N* (%)	No (*n* = 187) *N* (%)		
Perceived Usefulness	17.77 (2.42)	16.98 (2.51)	0.79 (0.18, 1.40)	2.558 **(0.011)**
Perceived Ease of Use	17.65 (2.30)	15.38 (2.96)	2.27 (1.59, 2.94)	6.627 **(<0.001)**
Social Influence	15.65 (3.69)	14.11 (3.12)	1.53 (0.72, 2.35)	3.707 **(<0.001)**

Bold values indicate significant association.

**Table 5:** Results of binary logistic regression models.

Model	Independent Variables	Standardized Beta Coefficients	*p*-Value	R^2^
1	PU	−0.150 *	0.048	0.204
	PEU	0.401 **	0.000	
2	PU	−0.165 *	0.035	0.208
	PEU	0.304 **	0.000	
	SI	0.042	0.351	

Dependent variable: virtual clinic use. * *p* < 0.05; ** *p* < 0.001.

## Data Availability

The data that support the findings of this study are available from the corresponding author upon reasonable request.

## Supplementary Materials

The following supporting information can be downloaded at https://www.mdpi.com/article/10.3390/healthcare10101893/s1, Supplementary file S1: Study Questionnaire—English version.
