# Supplementary material for: Awareness and Use of Virtual Clinics following the COVID-19 Pandemic in Saudi Arabia
Source: Healthcare (Basel). 2022 Sep 28;10(10):1893. doi: 10.3390/healthcare10101893 (PMC9601837; doi:10.3390/healthcare10101893)
Supplement: Supplementary file 1 [file healthcare-10-01893-s001.zip › healthcare-1891168-supplementary.pdf]

Virtual Clinics Questionnaire

1. **Are you aware of the presence of virtual clinics in Saudi Arabia?**
   - Yes
   - No

2. **Have you ever used virtual clinics before?**
   - Yes
   - No

3. **If you answered yes, please specify the name of the healthcare facility (or facilities) that has/have provided you with the virtual clinic service**
   …………………………………………………………………………………………………
   …………………………………………………………………………………………………

4. **How many times have you used virtual clinics?**
   - Once
   - Twice
   - More than 2 times

5. **In your last visit to the virtual clinic, what was the type of the healthcare facility?**
   - Private
   - Governmental

6. **In your last visit to the virtual clinic, what was the type of your visit?**
   - Initial consultation
   - Follow up

7. **In your last visit to the virtual clinic, who was the visit for?**
   - You
   - Someone else

8. **In your last visit to the virtual clinic, what was the clinic type?**
   - Family medicine
   - Internal medicine
   - Pediatric
   - Gynecology
   - Ophthalmology
   - Dermatology
   - Ear, nose, and throat surgery
   - Psychiatric
   - Neurology
   - Urology
   - Dentistry
   - Orthopedic
   - Other ……………….

9. **In your last visit to the virtual clinic, what device have you used?**
   - Mobile phone
   - Tablet (ex: iPad)
   - Laptop
   - Other………………

10. **In your last visit to the virtual clinic, what was the communication type?**
    - Voice call (only voice)
    - Video call (voice and picture)

11. **To what extent do you agree with the following statements?**

#	Statement	Strongly agree	Agree	Neutral	Disagree	Strongly disagree
1	Using virtual clinics would improve the quality of my health care.					
2	Using virtual clinics would improve my access to healthcare services.					
3	Using virtual clinics would save my time.					
4	Overall, I find virtual clinics highly useful.					
5	I would find that using virtual clinics is easy.					
6	I would find it easy for myself to interact with doctors using virtual clinics.					
7	Interacting with virtual clinics' systems would be clear for me.					
8	Overall, I would find that using virtual clinics is convenient.					
9	I like the idea of using virtual clinics.					
10	Using virtual clinics is a wise idea.					
11	Using virtual clinics would be a pleasant experience					
12	Utilizing virtual clinics is good.					
13	Relatives would support me to use virtual clinics.					
14	Friends would support me to use virtual clinics.					
15	Medical care personnel would support me to use virtual clinics.					
16	Media (socializing applications, social media influencers, news channels) would support the use of virtual clinics.					

12. What is your gender?
- Male
- Female

13. What is your nationality?
- Saudi
- Non-Saudi

14. What is your age?
- Less than 18
- 18-25
- 26-30
- 31-35
- 36-40
- 41-45
- 46-50
- 51-55
- More than 55

15. What is your marital status?
- Single
- Married
- Separated
- Widowed

16. In which province do you live?
- Eastern
- Riyadh
- Qassim
- Makkah
- Madinah
- Ha'il
- Al Jawf
- Tabuk
- Northern Borders
- Aseer
- Jizan
- Najran
- Al Baha

17. What is your education level?
- Primary school or less
- Intermediate school
- High school
- Diploma
- Bachelor’s degree
- Postgraduate degree

18. Do you have any chronic diseases?

- Yes
- No

19. What chronic diseases have you been diagnosed with (you can choose more than one answer)?

- Heart and cardiovascular diseases
- Hypertension
- Tumors
- Lung and respiratory diseases
- Diabetes
- Endocrine diseases
- Other……………….

20. What is your employment status?

- Employed
- Unemployed
- Retired

21. What is your income?

- Less than 5,000 SR
- Between 5,001 and 10,000 SR
- Between 10,001 and 15,000 SR
- Between 15,001 and 20,000 SR
- More than 20,000 SR

THE END
